# Are clinical delusions adaptive?

**DOI:** 10.1002/wcs.1502

**Published:** 2019-05-05

**Authors:** Eugenia Lancellotta, Lisa Bortolotti

**Affiliations:** ^1^ Philosophy Department and Institute for Mental Health University of Birmingham Birmingham UK

**Keywords:** adaptiveness, delusions, genetic fitness, wellbeing

## Abstract

Delusions are symptoms of psychiatric disorders such as schizophrenia and dementia. By and large, delusions are characterized by their behavioral manifestations and defined as irrational beliefs that compromise good functioning. In this overview paper, we ask whether delusions can be adaptive notwithstanding their negative features. Can they be a response to a crisis rather than the source of the crisis? Can they be the beginning of a solution rather than the problem? Some of the psychological, psychiatric, and philosophical literature has recently suggested that they can. We consider different types of delusions and different ways in which they can be considered as adaptive: psychologically (e.g., by increasing wellbeing, purpose in life, intrapsychic coherence, or good functioning) and biologically (e.g., by enhancing genetic fitness). Although further research is needed to map the costs and benefits of adopting and maintaining delusional beliefs, a more nuanced picture of the role of delusions in people's lives has started to emerge.

This article is categorized under:

Philosophy > Representation
Philosophy > Knowledge and Belief
Neuroscience > Cognition

## Graphical abstract

Delusions are considered to be the mark of madness in popular culture and one of the most severe symptoms of mental distress in clinical practice. In this overview, we acknowledge their adverse effects and raise the question whether they can ever be psychologically or biologically adaptive.

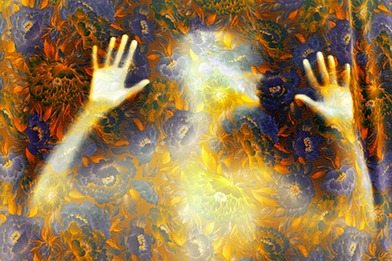

## 1 INTRODUCTION

In this paper we offer a brief overview of clinical delusions as they are discussed in the psychiatric, psychological, and philosophical literature, asking whether they can at the same time be symptoms of psychiatric disorders and have adaptive features. Delusions emerge in a variety of disorders, including schizophrenia, amnesia, dementia, delusional disorders, depression, and obsessive–compulsive disorders. They are typically unusual beliefs reported with conviction and held in the face of counterevidence. They can have varied content: people with delusions of persecution say that others want to harm them; people with delusions of mirrored‐self misidentification treat the reflection of their own face in the mirror as the face of a stranger, even though they preserve the general capacity to recognize images in the mirror as reflections.

Can delusions be adaptive? We take a trait or a mechanism to be adaptive if it contributes to the genetic fitness of the organism, cashed out in terms of increased chances for survival and reproduction. Physical and mental illness have been accounted for in terms of dysfunction (Wakefield, 1992) and adaptiveness (Murphy, 2005). One view is that illness arises either from the breakdown of a mechanism which no longer performs its function or from a change in the environment—some behavior that increased genetic fitness in the old environment no longer matches the needs of the organism in the new environment. Delusions are usually categorized as beliefs resulting from the breakdown of some adaptive mechanism (e.g., a deficit in the belief formation process) or as manifestations of behavior that contributed to genetic fitness in a different environment but no longer contributes to genetic fitness in the current environment (e.g., paranoia may have been adaptive in an environment where trusting others was especially risky). The notion of adaptiveness has been developed within evolutionary biology and in relation to genetic fitness, but by extension it has been used to characterize aspects of behavior that have significant benefits and whose value is not limited to an increase in an organism's chances of survival and reproduction. For instance, we can describe delusions as *psychologically* adaptive if they lead to increased wellbeing (McKay & Dennett, 2009) or have other psychological benefits such as promoting good functioning, enhancing meaningfulness, or restoring intrapsychic coherence.

First, we describe what delusions are (Section 2). Then, we consider how the notion of adaptiveness has been applied to them, and what the relationship may be between psychological and biological adaptiveness (Section 3). Further, we review arguments to the effect that some delusions have psychologically adaptive features (Section 4), and arguments to the effect that some delusions have biologically adaptive features (Section 5). Next, we introduce the case of delusions in obsessive–compulsive disorder (OCD) and in major depressive disorders (MDD), where recent evidence suggests that delusional beliefs can be biologically and psychologically maladaptive in the long term but psychologically adaptive in the short term (Section 6). In the end, we identify some areas for future research where new evidence may enable us to reach a more satisfactory conclusion about the adaptiveness of some delusions, while remaining skeptical about the possibility to establish whether delusions *in general* are adaptive (Section 7).

This skepticism is justified by the fact that “delusion” is used as a family‐resemblance term and that different conclusions about the adaptiveness of a delusion may be drawn depending not only on structural features that are shared by most delusions, but also on the content of the delusional beliefs, which is subject to great variation. The possibility that delusions are adaptive deserves further considerations notwithstanding the challenges we highlight, because it has significant implications for our conception of delusions as a symptom of psychiatric disorders and for our interactions with people reporting delusional beliefs.

## 2 WHAT ARE DELUSIONS?

There are some controversies about the best way to define delusions and there are also some debates about whether delusion overlaps with phenomena like self‐deception and confabulation. In this section we briefly address these issues.

Here are some definitions of delusions:

A false belief based on incorrect inference about external reality that is firmly held despite what almost everyone else believes and despite what constitutes incontrovertible and obvious proof or evidence to the contrary. The belief is not ordinarily accepted by other members of the person's culture or subculture (i.e. it is not an article of religious faith). When a false belief involves a value judgment, it is regarded as a delusion only when the judgment is so extreme as to defy credibility. (APA, 2013, DSM‐5, p. 819)

A person is deluded when they have come to hold a particular belief with a degree of firmness that is both utterly unwarranted by the evidence at hand, and that jeopardises their day‐to‐day functioning. (McKay, Langdon, & Coltheart, 2005, p. 315)

Delusions are generally accepted to be beliefs which (a) are held with great conviction; (b) defy rational counter‐argument; and (c) would be dismissed as false or bizarre by members of the same socio‐cultural group. (Gilleen & David, 2005, pp. 5–6)

The definitions above characterize delusions on the basis of their *surface features*, and in particular their negative epistemic features, where “epistemic” denotes something that has to do with knowledge and belief. Delusions are defined as beliefs that are *unwarranted*, *fixed*, *resistant to counterargument*, and *implausible*. Some definitions also include negative psychological features, adding that delusions typically compromise wellbeing or good functioning. Most definitions make no reference to the mechanisms responsible for the formation of delusional beliefs. This is not surprising as delusions emerge in a variety of contexts and thus researchers have not come to a consensus on the best etiological account of delusions.

Delusions used to be divided into *functional* and *organic*: a delusion was called *organic* if its presence was explained by brain damage (such as injuries affecting the right cerebral hemisphere), and *functional* if its presence was explained by psychodynamic or motivational factors. Today, it is acknowledged that there are biological and psychological factors contributing to most types of delusions, although there may be still some work to do in order to identify such factors with precision and map the interaction between them.

Consider the two delusions we introduced earlier, persecution and mirrored‐self misidentification. Persecution is usually a *polythematic* and *elaborated* delusion, that is, it extends to more than one theme where the themes can be interrelated, and it is well integrated in the person's belief system, often driving action that is consistent with the person believing the content of the delusion. For instance, a young woman who believes that she is surrounded by alien forces that control her own actions and are slowly taking over people's bodies might decide to run away to protect her loved ones from danger (Payne, 2013).

Mirrored‐self misidentification is usually *monothematic* and *circumscribed*. That means that, apart from the content of the delusion itself, the person may not make any other implausible claim and the delusion may not be supported by, or support, the person's other beliefs. Suppose an elderly man experiencing mirrored‐self misidentification is asked whether “the stranger in the mirror” resembles him. The man might acknowledge that the stranger looks like him, which makes his delusional belief that the person in the mirror is a stranger even less plausible (Breen, Caine, Coltheart, Hendy, & Roberts, 2000).

Other examples of monothematic delusions are Capgras and Cotard. In Capgras the person claims that a dear one (a close relative or the spouse) has been replaced by an impostor. In Cotard the person reports being disembodied or dead. In most cases such beliefs are not interacting with the person's other beliefs, although they may be defended with reasons when challenged. So, the person with Capgras may not go looking for the loved one who is believed to be missing (although some people with Capgras are hostile and even violent towards the alleged impostor) and the person with Cotard may not act dead (although some people with Cotard may stop routine behavior such as bathing).

One might wonder what the difference is between delusion and other false or irrational behaviors we find in the clinical and nonclinical population. Most definitions of delusions fail to provide a set of necessary and sufficient conditions for the phenomenon and leave it open that delusions may overlap with, for instance, cases of self‐deception and confabulation.

Self‐deception is much more common than delusion and does not seem to have the same disruptive consequences on functioning. In some instances, the phenomena cannot be easily distinguished, as they both involve some belief that is not well‐grounded and that people nonetheless report with conviction and are often prepared to act upon. The distinction between delusion and self‐deception is often characterized by the role of *motivational factors*. In self‐deception, motivational factors are central. If we are self‐deceived, we end up believing what we *want to* be the case (“My partner is faithful to me,” “I failed the exam because I was too tired after a night out,” etc.). In delusion, motivational factors may have a role, but need not. The content of delusional beliefs is not always something we find desirable. In some delusions, we might conjure a positive image of ourselves, for instance, as people who were chosen by God to accomplish an important mission in delusions of reference; as the only people able to understand a complex conspiracy in delusions of grandeur; or as attractive sexual partners pursued by famous people in erotomania.

In other delusions, though, we see ourselves as overwhelmed by guilt (delusions of guilt), or manipulated by external forces who can control our actions or even insert thoughts in our heads (delusions of passivity and thought insertion). That said, even thoroughly unpleasant delusions, such as delusions of persecution, might have a defensive role to play, protecting our self‐esteem. Bad events in our lives are explained by the evil intentions of the persecutors and not by our own failings. Typically, “delusion” refers to a clinical phenomenon and “self‐deception” to a nonclinical one, however, there is no sharp distinction between delusion and self‐deception. In this article we shall consider some delusions that are described as “motivated” in that they represent the person's reality as more pleasant than it is. In those cases, the line between delusion and self‐deception cannot be clearly drawn as motivational factors are likely to play a role in both phenomena.

Another distinction that is not made consistently in the literature is between delusion and confabulation. Confabulation, just like delusion, is primarily characterized by negative epistemic features—it is described as an ill‐grounded belief or a false narrative (e.g., Hirstein, 2005). It is key to confabulation that the belief or narrative is genuinely endorsed and presented with no intention to deceive. So, there is a substantial overlap with delusion. The main difference between delusion and confabulation is that, as a clinical phenomenon (*narrow confabulation*), confabulation concerns the distortion or fabrication of a memory, and thus it most often emerges in psychiatric disorders that feature serious memory impairments such as amnesia and dementia. Delusion does not need to involve memory distortions or fabrications.

As a nonclinical phenomenon (*broad confabulation*), confabulation is just an explanation that is ill‐grounded (e.g., Hirstein, 2005; Nisbett & Wilson, 1977), and thus it does not need to involve any memory impairment. That said, some differences can be found also between delusion and broad confabulation. Whereas a delusion is usually an implausible belief that can be further elaborated but does not need to, a confabulation can be very plausible and often takes the shape of an explanation or a narrative, thus exhibiting a high level of elaboration and integration with the person's other beliefs.

An influential answer to the question about the differences between delusion and self‐deception, or delusion and broad confabulation, is that the former is a pathological phenomenon that characterizes a clinical population, whereas the latter is a widespread phenomenon in the nonclinical population that does not need to impair functioning and does not count as a symptom of mental distress. However, we should be cautious and avoid relying too heavily on the distinction between *pathological* and *nonpathological* belief or between *clinical* and *nonclinical* populations, because it is not clear what makes a belief pathological and beliefs with the same epistemic features as delusions are to be found in people who do not experience mental distress and do not attract a psychiatric diagnosis (Bortolotti, Gunn, & Sullivan‐Bissett, 2017).

## 3 ADAPTIVENESS

The adaptiveness of delusions has been briefly explored in the recent literature, and we shall review some of the identified costs and benefits of adopting or maintaining delusional beliefs in Sections 4, 5, and 6. Here we explain why we distinguish such costs and benefits in biological and psychological, and consider some hypotheses about what the relationship between the two categories may be.

“Adaptiveness” is first and foremost a biological notion. The goal of adaptive traits is to support the reproductive success and survival of the biological organism they belong to. Crucially, adaptiveness is not a timeless process but rather a historical one, and an adaptive trait is closely connected to the environment in which it develops. Thus, some traits can be adaptive in one environment without being adaptive in other environments, or they can lose their adaptiveness as a consequence of environmental changes. By analogy with biological adaptiveness, some authors speak of *psychological* adaptiveness when a belief, state of mind. or mechanism delivers important psychological benefits to an organism, such as enhancing its psychological wellbeing.

It is not clear how biological and psychological adaptiveness relate to each other. As Ryan McKay and Daniel Dennett eloquently write, behaviors that are psychologically adaptive may not be biologically so, and behaviors that are biologically adaptive may not be psychologically so:

Here we must be careful to honour a distinction, often complacently ignored, between human happiness and genetic fitness. If the most promising path, on average, to having more surviving grand offspring is one that involves pain and hardship, natural selection will not be deterred in the least from pursuing it (it is well to remind ourselves of the insect species in which the males are beheaded in the normal course of copulation, or – somewhat closer to home – the ruthless siblingcide practiced by many bird species). (McKay & Dennett, 2009, p. 502)

As Randolph Nesse (1998, p. 401) also says: “natural selection shaped the regulation mechanisms for maximal reproductive success, not for peace and happiness.” In some cases, nature seems to have designed us to derive pleasure from those behaviors which foster reproductive success and survival, such as sexual intercourse (e.g., Fleischman, 2016). However, in other cases, biological and psychological adaptiveness come apart. For instance, enjoying very low levels of anxiety can be psychologically beneficial, but poses a threat to reproductive fitness. This is because, if we underestimate danger, then we drastically decrease our chances of survival and reproduction (e.g., Lee, Wadsworth, & Hotopf, 2006). In the case of beliefs more specifically, as McKay and Dennett (2009) argued, a belief leading to psychological wellbeing may or may not be biologically adaptive. We will review cases of delusions which seems to be both psychologically and biologically beneficial in the short term, such as motivated delusions, and cases of delusions where psychological benefits come with an increase of the severity of symptoms, such as delusions in MDD and OCD.

### 3.1 The doxastic shear‐pin

What would it mean for delusions to be adaptive? As delusions are commonly defined on the basis of their negative features and are symptoms of a number of severe disorders, the possibility that they are adaptive in any sense is often quickly dismissed. However, it has been suggested that delusions are an instance of misbelief that is potentially adaptive. McKay and Dennett introduce the metaphor of the *doxastic shear pin* to ask whether delusions are the outcome of a mechanism that is designed to malfunction in some circumstances in order to prevent a more serious malfunctioning from occurring.

A shear pin is a metal pin installed in, say, the drive train of a marine engine. The shear pin locks the propeller to the propeller shaft and is intended to “shear” should the propeller hit a log or other hard object. (McKay & Dennett, 2009, p. 497)

Shear pins are designed to break under extreme conditions, allowing a system to keep functioning, although in an imperfect manner. Delusions might act as shear pins, allowing the doxastic system to keep working in times of extreme distress. In this picture, delusions emerge as a response to a breakdown threatening a person's epistemic and emotional engagement with reality.

We envision doxastic shear pins as components of belief evaluation machinery that are “designed” to break in situations of extreme psychological stress (analogous to the mechanical overload that breaks a shear pin or the power surge that blows a fuse). Perhaps the normal function (both normatively and statistically construed) of such components would be to constrain the influence of motivational processes on belief formation. Breakage of such components, therefore, might permit the formation and maintenance of comforting misbeliefs – beliefs that would ordinarily be rejected as ungrounded, but that would facilitate the negotiation of overwhelming circumstances […] and that would thus be adaptive in such extraordinary circumstances. (McKay & Dennett, 2009, p. 501)

McKay and Dennett consider the possibility that some delusions may be biologically adaptive as doxastic shear pins, and in the end they reject this hypothesis. However, this has not stopped other scholars using the doxastic shear pin metaphor to argue for the potential adaptiveness of delusions. For instance, Lisa Bortolotti argued that motivated delusions, as well as delusions in schizophrenia, can have both *psychological* and, indirectly, *epistemic* benefits (Bortolotti, 2015, 2016). By enabling the doxastic system to continue working (albeit imperfectly) when the person is at risk of being overwhelmed by negative emotions or by the uncertainty caused by anomalous experience, delusions support the person's capacity to pursue and achieve some of her epistemic goals. Without the psychological relief that the delusion offers, the person's capacity to pursue and achieve her epistemic goals would be compromised by a disrupted or absent interaction with her surrounding environment.

Philip Corlett and colleagues argue against McKay and Dennett that the doxastic shear‐pin hypothesis leads to the claim that all delusions are *biologically*—not just psychologically—adaptive (see Fineberg & Corlett, 2016; Mishara & Corlett, 2009). This is because on their account delusions arise as a response to aberrant prediction error signals and permit “ongoing function in the face of paralyzing difficulty” (Fineberg & Corlett, 2016, p. 73). Fineberg and Corlett argue that the function of delusions is to keep a person in contact with her environment in situations of emergency and view this as a contribution to survival: “our model indicates how delusions may be adaptive as a shear pin function by enabling the patient to remain in vital connection with his/her environment” and “patients continue to respond reflexively to the environmental cues incumbent upon them, necessary for continued survival” (Mishara & Corlett, 2009, p. 531). On this account, the emergency is described differently from how it is described by McKay and Dennett. For McKay and Dennett, the risk is that negative emotions may overwhelm the person and cause a complete absence of interaction with the environment via severe depression, which may occur when the shear pin does not break. For Mishara and Corlett, instead, the risk is that the learning system crashes due to the persistent prediction‐error signals it receives and is disabled, leaving the person unable to gather information about aspects of her environment that are crucial to her survival. So, the averted danger is presented not just as a psychological benefit, but as a biological one.

We will come back to this account in the next section, where we consider the hypothesis that delusional beliefs are biologically adaptive.

## 4 BIOLOGICAL ADAPTIVENESS OF DELUSIONS

In this section, we review the reasons for considering delusions maladaptive and then turn to one influential argument for the biological adaptiveness of delusions, suggesting that they are instrumental to learning being restored after the effect of disrupting prediction error signals. There is evidence suggesting that delusions are biologically maladaptive. The presence of delusions is a predictor of poor long‐term outcome in people with paranoid psychosis (Jørgensen, Aagaard, Jespersen, & Mortensen, 1987) and people with schizophrenia who often experience hallucinations and delusions marry and reproduce less than controls (Nanko & Moridaira, 1993), suggesting that psychiatric disorders characterized by psychotic symptoms compromise a person's chances to mate and reproduce. Although there is still disagreement about how delusions are formed and maintained, the plausible candidate theories present delusions as the outcome of a malfunctioning mechanism, though the nature of the mechanism involved varies from one account to the next. One‐factor theories (Maher, 1974) and predictive processing models (Corlett, Taylor, Wang, Fletcher, & Krystal, 2010; Fletcher & Frith, 2008) argue that abnormal perceptions or aberrant predictive error signals are necessary and sufficient for delusional beliefs. Instead, two‐factor accounts (Coltheart, Menzies, & Sutton, 2010; Davies, Coltheart, Langdon, & Breen, 2001; Stone & Young, 1997) hold that abnormal data are necessary but not sufficient to account for the adoption of delusions, introducing a second factor. This is to be identified with some form of reasoning bias or deficit. Error management theorists argue that delusions are the outcome of extreme, pathological versions of evolutionary biases (Miyazono, 2015; McKay & Dennett, 2009, p. 502). What theory of delusion formation we adopt matters to whether we believe that delusions can be adaptive. Delusions are biologically adaptive if, as a response to a crisis of some sort (anomalous perception or overwhelming distress), they enhance a person's chances of reproductive success and survival by conferring systematic biological benefits. The view that delusions have biological benefits has been defended by some predictive coding theorists and we now turn to their account.

### 4.1 Making sense of anomalous experience

Predictive coding assumes that the brain is a predictive machine governed by a simple principle: to minimize uncertainty and to build an internal model of the world which reduces to a minimum the discrepancy between what is expected and what is experienced (Hohwy, 2014). It proves difficult to reduce predictive coding to either a one‐factor or a two‐factor account (Miyazono, Bortolotti & Broome, 2015). Contrarily to both one and two‐factor theories, prediction error theories do not draw a sharp distinction between perception and belief (Fletcher & Frith, 2008; Williams, 2018). This is because in predictive coding perception and belief are two temporal phases of the same process of hierarchical Bayesian inference. While perceptions take place lower down in the hierarchy, at a faster and more limited spatiotemporal scale, beliefs (delusions included) are generated at a higher level of the same hierarchy, at a larger and more abstract spatiotemporal level. Another important distinction between prediction‐error theories and either the one‐factor or the two‐factor account is that, instead of perceptions unidirectionally influencing beliefs, in predictive processing perceptions and beliefs dynamically sculpt one another, so that we do not just believe what we see but we also see what we believe (McKay, 2012).

According to prediction error theories, delusions are reached via a single impairment in the hierarchy of inference (Bortolotti & Miyazono, 2015). The impairment in question would be represented by aberrant prediction‐error signals, which cause the subject to depart from ideal Bayesian norms of rationality. Normally, prediction errors signal a discrepancy between what the person expects and what she actually experiences, so that the experience either is discarded because it conflicts with the person's existing beliefs, or it is taken to imply that something was amiss in the person's expectations and new beliefs need to be adopted. The criteria according to which an experience either is discarded or causes prior beliefs to be updated depend on the estimation of the precision of the prediction error. If a prediction error is estimated to be precise, it results into an updating of the existing beliefs; if it is not estimated to be precise, the experience is discarded.

In people with delusions, prediction errors are elicited when they should not, by events that appear significant and salient but are unsurprising, or they are mistakenly weighted as highly precise.

[P]rediction error theories of delusional formation suggest that under the influence of inappropriate prediction error signal, possibly as a consequence of dopamine dysregulation, events that are insignificant and merely coincident seem to demand attention, feel important and relate to each other in meaningful ways. Delusions ultimately arise as a means of explaining these odd experiences.” (Corlett et al., 2010, p. 1)

Due to the high precision erroneously assigned to the prediction error, its rejection is not a viable option. Hence a revision of priors takes place, and a delusional belief is adopted as a new prior making sense of the anomalous experience. At a later stage, further aberrant perceptions are explained in the light of the delusional prior, giving way to a process of reinforcement which strengthens the endorsement of the delusion (Fineberg & Corlett, 2016, p. 79).

### 4.2 Resuming automated learning

Within a predictive coding framework, advocates of delusional adaptiveness argue that the main biological benefit of delusions consists in enabling the learning system to resume its normal function in the face of aberrant perceptions and prediction‐error signals (Fineberg & Corlett, 2016, pp. 73–76; Mishara & Corlett, 2009, p. 530). By explaining anomalous perceptions and prediction errors, delusions not only provide some short‐term relief from uncertainty, but they also salvage the learning capabilities of the person by protecting her from the danger of having frequent, unexplained and highly precise aberrant prediction error signals. These are thought to disrupt the normal process of learning and, as far as they persist, to severely impair the contact of the person with the world. The view is that, by explaining aberrant prediction errors, delusions prevent a complete loss of contact with reality. This is regarded to be biologically adaptive, because being in contact with reality in an impaired manner affords a person better chances of survival than being in no contact with reality at all.

Although on this view delusions “rescue” the learning system, when adopted and maintained, they disable its most flexible and cognitively expensive part. There are in fact two distinct parts of the learning system which compete to control behavior, one goal‐directed, the other habitual. While the former “involves learning flexible relationships between actions and outcomes” (Mishara & Corlett, 2009, p. 530), the latter mirrors a more stereotyped relationship between stimuli and responses, such that a particular stimulus always triggers a particular response, independently from the outcome. When delusions are adopted, the flexibility of the former system is disabled to permit ongoing learning, while the latter system takes over in controlling the person's delusional beliefs (Fineberg & Corlett, 2016, p. 76). On the account, this explains the fixity and inflexibility of delusional states, features that make them more similar to habits than to actions that are sensitive to rewards. Although the goal‐directed system still functions in the formation and assessment of other beliefs, it is disabled with regard to the delusional beliefs: it would be cognitively too costly to keep it working in the face of anomalous perceptions and predictive errors.

Here is an example. The belief that her spouse has been replaced by an identically looking imposter provides an explanation for the anomalous feeling of unfamiliarity the person looks at experiences when she sees her spouse's face (*delusional formation*). The delusional belief (“My spouse has been replaced by an impostor”) is then reinforced by the persistency of the aberrant feelings of unfamiliarity and ends up being adopted as a new prior which explains the perceptions in question (*delusional maintenance*). There are several advantages to entertaining such a bizarre belief: the discrepancy between perceptions and expectations is reduced, the anxiety induced by the unexplained experiences is relieved, and learning is resumed as soon as the prediction errors are got rid of. Now the person can focus her attention on other things rather than spending all her resources trying to make sense of a persistent feeling of unfamiliarity when looking at her spouse. Life can go on, but at a price.

The sensitivity of the new belief to counter‐evidence has been disabled. From a cognitive point of view, it would in fact be too expensive to maintain belief flexibility in the presence of the mental distress brought about by the aberrant prediction‐error signals which accompany the delusional perception (Fineberg & Corlett, 2016, p. 76; Gold et al., 2013). Hence, delusions restore the contact of the person with reality—in the sense that they allow the person to focus her attention and mental energy on something different from her aberrant perceptions—but at the cost of the responsiveness of her newly formed beliefs to evidence.

### 4.3 Problems with the adaptiveness claim

How plausible is it that delusions contribute to keep a person in contact with reality? The idea is that delusions provide an explanation to previously unexplained aberrant perceptions that would otherwise monopolize the person's cognitive resources. By doing so, delusions would allow the person to focus on aspects of the world other than those that are the object of her aberrant perceptions. In this picture, the adoption of a delusion restores the process of learning which is momentarily blocked by the presence of salient, unexplained perceptions.

This account is controversial. Many delusions are thought to absorb and hijack cognitive resources instead of freeing them up. As we saw earlier, people with delusions of persecution may be preoccupied that ill‐intentioned aliens wish them harm and that thought might dominate their mental life. They often take action like moving cities and avoiding contact with people to keep themselves and others safe. It is apparent that in such cases delusions are time and energy consuming rather than cognitively liberating. However, a supporter of the adaptiveness of delusions might not need to deny that maintaining the delusions in the long run can cause severe disruption to a person's life. Rather, the claim is that, when the delusion is adopted, the person may be better off by believing that alien forces are persecuting her than being bombarded by inexplicable and discomforting perceptions. The veracity of such a claim is an empirical issue, as it implies ascertaining whether people with abnormal perceptions and no delusions are worse off than people having both abnormal perceptions and delusions. One consideration is that the phase of abnormal perceptions usually preceding the formation of delusions (e.g., the so‐called “prodromal phase” in schizophrenic delusions) might last days, months, even years (Mishara & Corlett, 2009). If someone experiences aberrant perceptions for years, it is implausible to imagine that she is in a state of emergency for such a long time. Again, a supporter of the adaptiveness of delusions might reply that the shear pin breaks only when aberrant perceptions have reached a certain threshold of intensity, and this might well take years to happen.

## 5 PSYCHOLOGICAL ADAPTIVENESS OF DELUSIONS

In this section we consider the rather obvious psychological costs of adopting delusional beliefs and the less obvious (often short‐term) benefits that adopting delusions can bestow on agents who are already experiencing what can be described as a crisis (McKay & Dennett, 2009). Many delusions have detrimental psychological effects, causing unhappiness and worry to the people who experience them (see, e.g., Garety & Hemsley, 1987). All delusions can be disruptive in the sense that they alter the person's sense of reality and compromise socialization by causing the person with the delusions to become overly preoccupied with the content of the delusion and isolated. Moreover, some psychological harm can stem directly from the content of the delusion. We saw earlier that in some cases of persecutory delusions, the person lives in fear, worried that the threats she experiences are going to cause her or her loved ones pain or death.

Some other delusions, however, are not held with distress. On the contrary, depending on their content, some delusions can confer a sense of purpose and boost self‐esteem (see, e.g., McCreery & Claridge, 2002; Peters, Day, Mckenna, & Orbach, 1999; Roberts, 1991). For instance, delusions of reference and delusions of grandeur can make the person feel important and worthy of admiration. Whether the delusions with desirable content have positive or negative psychological effects depends on the preferred notion of wellbeing, as has been argued before (for instance, see Miyazono, 2015). Delusions of reference and grandeur have psychological benefits on a *hedonic* view of wellbeing, intended as a subjective feeling about how well one's life is going. On a *eudaimonic* view of wellbeing, however, where the authenticity and meaningfulness of a person's life, her agency, and her capacity to function socially are also taken into account, even delusions with positive content can have negative effects. Believing that one is Napoleon is empowering, but the clash between the belief and the reality of the person's surroundings means that there is a loss of contact with the physical and social environment, and alienation ensues.

That said, there are accounts where delusions are described as responses to a critical situation, maybe the beginning of a solution, rather than the source of the problem. It is not clear to what extent this view can be generalized to all types of delusions, but some adaptive features can be identified in different types of delusions. Some delusions in schizophrenia provide temporary anxiety relief by offering explanations for unusual experiences. Some elaborated delusions can increase a sense of meaningfulness and purpose. Some “motivated” delusions make a harsh reality temporarily less distressing. And some delusions in dementia fill gaps in a person's failing understanding of the world.

### 5.1 Putting an end to uncertainty

In some influential accounts of the formation of delusions in the context of schizophrenia (such as the ones provided by Kapur, 2003), the adoption of a delusional belief is said to offer the person some relief from anxiety:

First, endogenous psychosis evolves slowly (not overnight). For many patients it evolves through a series of stages: a stage of heightened awareness and emotionality combined with a sense of anxiety and impasse, a drive to ‘make sense’ of the situation, and then usually relief and a ‘new awareness’ as the delusion crystallizes and hallucinations emerge. (Kapur, 2003, p. 15)

The idea is that in the first stage of psychosis the person has experiences that appear to her as inexplicably salient and this causes anxiety and negative emotions that can become overwhelming, with adverse effects for well‐being. The sense is that something important is about to happen, but there is no indication of what that something might be. As in the classic literature on delusions by Jaspers (1963) and Conrad Mishara (2010), the delusion emerges as an explanation or a revelation, taking away the sense of uncertainty. Although the adoption of the delusion can be seen as a temporary relief from anxiety, the delusion itself can become a source of anxiety in the long run. Especially when delusions have distressing content, they can affect negatively a person's life. That is why anxiety relief is likely to be temporary, an effect of the *adoption* but not of the *prolonged maintenance* of the delusion.

People may no longer feel anxious about their hypersalient experience (for example, ‘how should I interpret this?’), but they can feel anxious and distressed about how the world is according to the delusion (for example, ‘how can I escape from the alien forces persecuting me?’). (Bortolotti, 2016, pp. 890–891)

### 5.2 Finding life meaningful

In an interesting study aimed at exploring the potential adaptiveness of delusions (Roberts, 1991), people with elaborated and systematized delusions were found to score higher than people in remission, rehabilitation nurses, and Anglican ordinands in the “purpose in life” test and the “life regard” index, which measure respectively a person's experience of meaning and purpose in life and a person's regard for her own life. The conclusion of the study is that “for some there may be satisfaction in psychosis and that [delusion formation] is adaptive” (Roberts, 1991, p. 19). The psychological adaptiveness of the delusion is ascribed to two factors: the delusion serves as an explanation for an experience that was distressing or perplexing for the person, bringing relief from uncertainty; and the delusional reality may be preferable to the actual reality the person finds herself in, playing a defensive function.

More recently, it has been found that people in acute delusional state may have a greater “sense of coherence” than people with no psychiatric diagnosis (Bergstein, Weizman, & Solomon, 2008). The sense of coherence encompasses the feeling that there are projects that are both worth engaging and challenging, and that the person has the resources to pursue them. The sense of coherence is positively correlated with wellbeing.

### 5.3 Overcoming trauma

In Section 3, we saw that one of the main reasons put forward in the literature for the psychological adaptiveness of delusions—McKay and Dennett's original doxastic shear pin account—is that some delusions (especially so‐called *motivated* delusions) play a defensive function, representing the world as the person would like it to be.

One example is anosognosia, which most commonly involves the denial that a limb is paralyzed (“I am moving my arm,” when the arm cannot move; or “I can climb stairs but I am a little slow,” when legs are paralyzed). People with anosognosia often deny obvious evidence of their impairment (Berti, Spinazzola, Pia, & Rabuffetti, 1993, p. 164). The delusion has negative effects, compromising the shared reality between the person and her closest ones. Further problems are likely to emerge if the person refuses to acknowledge the implications of her impairment and fails to engage in rehabilitation.

However, when people need to live with the consequences of trauma, denying the impairment can be adaptive in the short term (Fotopoulou, 2008; Ramachandran, 1996) as people do not have to accept that they are severely disabled and that their lives have dramatically changed. Overwhelming negative emotions would compromise the person's capacity to interact with her physical and social environment because severe depression would not be conducive to the pursuit of her goals. Indeed, people who experience anosognosia are found to have fewer negative emotions and reduced anxiety with respect to people who acknowledge their impairment.

### 5.4 Filling gaps

Delusional beliefs may emerge in people with middle‐ to late‐stage dementia, usually beliefs involving suspicions or paranoia (e.g., the belief that someone is stealing things). Although the memory impairment can be a trigger for the suspicion or the accusation (e.g., the person cannot remember where she placed her possessions), such delusional beliefs are not distorted memory reports but firmly held beliefs that the person does not typically abandon as a result of a reasoned argument.

Such delusional beliefs are extremely distressing for caregivers who are often the object of suspicions and accusations, and who need to manage the person's delusions‐driven behavior. However, in the circumstances in which the person finds herself, it is not difficult to see how the delusional beliefs play the role of (implausible but) handy explanations for unexpected events (“I cannot find my wallet”), thereby offering some short‐term relief to them as they are trying to make sense of a physical and social world that is increasingly hard to navigate.

## 6 DELUSIONS IN OCD AND MDD

Here we turn to delusional beliefs emerging in disorders that traditionally do not involve psychotic symptoms, such as OCD and MDD. Although the question whether delusions are adaptive in these contexts may be premature, the literature suggests that there may be some short‐term psychological benefits for the people with OCD or MDD who develop delusional beliefs congruent with their habits or moods.

### 6.1 Delusions in OCD

According to the *Diagnostic and Statistical Manual of Mental Disorders*,

OCD is characterized by the presence of obsessions and/or compulsions. Obsessions are recurrent and persistent thoughts, urges, or images that are experienced as intrusive and unwanted, whereas compulsions are repetitive behaviours or mental acts that an individual feels driven to perform in response to an obsession or according to rules that must be applied rigidly. (APA, 2013, DSM‐5, p. 235)

Most people with OCD display good insight into their illness, regarding their obsessive–compulsive behaviors as unwarranted and exaggerated. However, in a minority of cases (around 4% according to DSM‐5, p. 238) people hold their obsessive–compulsive thoughts with delusional conviction, fearing that not ritualising will bring about dreaded consequences.

[X] thought a supernatural ‘power’ inserted unpleasant thoughts into her mind, e.g. “if you read that book a relative will die” […] To appease the ‘power’ and the thoughts, she developed complex counting rituals pervading her daily activities. She also did ritualistic hand‐washing and checking. She avoided specific numbers, colours and clothes and counted from 0 to 8 on her fingers and toes throughout the day […] She felt unable to resist the rituals, as her belief in negative consequences was absolute. (O'Dwyer & Marks, 2000, p. 282)

The reasons why obsessions might develop into delusion‐like beliefs are far from being fully understood; however, some proposals have been advanced. It has been hypothesized that high levels of anxiety, combined with obsessions, predict the formation of delusional beliefs (Bortolon & Raffard, 2015). Single‐themed obsessions, magical thinking, and depressive symptoms are all factors that contribute to delusional ideation in OCD on some accounts (Fear, Sharp, & Healy, 2000). Further studies are required to explore if delusional experiences in OCD might play an adaptive role. Is delusional ideation in OCD an adaptive response or just a worsening of the distress which characterizes obsessive–compulsive thoughts and behaviors?

While the latter hypothesis has garnered most of the attention from research—with studies showing that delusional forms of OCD are associated with “a graver clinical picture” than nondelusional forms (Poyurovsky, 2013, p. 173)—so far the former has not received much consideration. However, an adaptive role, whether biological or psychological, for delusional forms of obsessions should not be prematurely ruled out. Delusional beliefs in OCD might in fact help the person preserve a sense of rational agency. An agent is deemed to be rational when her actions are consistent with her desires and beliefs. For example, the act of Jamie's drinking a glass of water is rational if it results from his desire to extinguish his thirst and the belief that drinking a glass of water will effectively do so.

In OCD desires are expressed by obsessions. For example, Bonnie might strongly want her house not to burn down (*obsession/desire*) and, in order to prevent that, she might think that she has to check the stove 30 times per day (*belief*). As a consequence of her desires and beliefs, Bonnie will feel driven to perform the action of checking the stove 30 times per day (*action/compulsion*). Even if people with OCD do not assume that the link among their obsessions, beliefs, and actions is a rational one, they still feel the drive to perform the relevant actions. In other words, Bonnie might not be convinced that checking the stove 30 times per day is necessary to prevent her house from burning down, yet she performs that action anyway. This generates problems for Bonnie's sense of rational agency: people with OCD cannot often make sense of their own actions, feel out of control, and are conflicted due to the discrepancy between their beliefs and actions. If an obsession reaches delusional intensity, however, the discrepancy between what is believed and what is acted upon significantly diminishes. If Bonnie is (delusionally) convinced that checking the stove 30 times per day will prevent her house from burning down, then her action—checking the stove—will seem more rationally justified to her than if she lacked that conviction. Arguably, the preservation of one's sense of rational agency confers a sense of control on one's actions and a feeling that one's actions effectively bring about the satisfaction of one's desires. Hence, it might be the case that, even if biologically maladaptive, delusions in OCD confer psychological benefits that come from restoring the intrapsychic coherence necessary for preserving one's sense of rational agency.

### 6.2 Delusions in MDD

Delusions in MDD do not appear to be adaptive in a biological sense, as people with depression who also have delusions show a higher suicide rate than their nondelusional counterparts (Roose, 1983), suggesting that the presence of delusions can impact negatively on survival. However, a similar argument as for delusions in OCD can apply to delusions in MDD. The presence of delusions can reduce the perceived conflict between the agent's mood and the agent's beliefs, which can be psychologically draining.

So‐called *psychotic depression* or *depressive psychosis* is a major depressive episode that can be accompanied by delusions and hallucinations (Gerretsen et al., 2015; Hales & Yudofsky, 2003) as well as very low mood, lack of interest in everyday matters, problems with sleeping, feelings of guilt, and obsessive self‐accusations. Most delusions in severe depression are *mood‐congruent*, which means that their content matches the person's mood experienced (Hales & Yudofsky, 2003). According to the *Diagnostic and Statistical Manual of Mental Disorders* (APA, 2013, DSM‐5), common themes of depressive delusions are persecution, guilt, punishment, personal inadequacy, or disease.

It is controversial whether depression with delusions is a form of unipolar depression or a form of psychosis (e.g., Frances, Brown, Kocsis, & Mann, 1981; Parker, Roy, Hadzi‐Pavlovic, & Pedic, 1992). Those who argue that depression with delusions is a form of depressive disorder (e.g., Stanghellini & Raballo, 2015) point to a significant difference between delusions in schizophrenia and delusions in depression: whereas delusions in schizophrenia are a revelation and disclose some *new and surprising* content, the content of delusions in depression contains information that is *already known* and *familiar*. For instance, delusions of guilt in MDD may validate a feeling of guilt and confirm the person's conviction that she has done something wrong.

Following from this observation and drawing on an influential theory of depression (see Beck, 1967), it has been argued that delusions in depression can be viewed as adaptive from a psychological point of view (see, e.g., Antrobus & Bortolotti, 2016). People with MDD have feelings that are often not confirmed by the evidence available to them; typically, they feel guilty and inadequate. Delusions that contain negative self‐related information help them preserve coherence in their self‐appraisals. This is a significant effect, because we already know that people have a general tendency to restore coherence between moods and beliefs, or between conflicting beliefs, when coherence is compromised (see for instance, Heider, 1946; Festinger, 1957). It is possible that mood‐congruent delusions in MDD offer validation for the guilt, shame, and hopelessness the person experiences, and contribute to avoiding fragmentation in one's self‐concept, enhancing intrapsychic coherence. At the same time, they may cause depressive symptoms to become more severe, thus compromising biological adaptiveness overall.

## 7 HOW DO WE ESTABLISH AND MEASURE ADAPTIVENESS?

It is controversial whether delusions can be biologically adaptive, but it is possible to describe delusions as psychologically adaptive, in some cases and in the short term. The psychological benefits of delusional beliefs vary depending on the type of beliefs, their content, and the context in which they are adopted. This means that different measures of psychological adaptiveness will apply to different delusions. The notions of subjective wellbeing and psychological wellbeing capture key aspects of the philosophical accounts of wellbeing known respectively as *hedonism* and *eudemonism*. Hedonism holds that wellbeing is a subjective feeling of pleasure and pain. Instead, according to eudemonism, wellbeing also depends on a positive assessment of one's functioning and on the meaningfulness of one's life. Both parameters are relevant to the psychological adaptiveness of delusions.

To sum up, we saw that some delusional beliefs are thought to relieve anxiety and stress; the effects of delusions that are elaborated may include enhancing the sense that one's life is important and meaningful; motivated delusions can be described as a temporary coping mechanism in response to trauma or adversities; delusional beliefs in dementia can help fill explanatory gaps that would be otherwise difficult to live with, enabling one to build a coherent self‐image encompassing beliefs, emotions, and behaviors; and finally, delusions can sustain the sense of one's rational agency, restoring intrapsychic coherence. In most of our examples, delusions are thought to make a positive contribution to the hedonic form of wellbeing, which can be measured relying on self‐reports or behavior (e.g., by asking people how their lives are going) or on physiological indicators (e.g., by tracking the oscillations in the levels of stress‐related hormones such as cortisol as in Fineberg & Corlett, 2016).

It is more difficult to ascertain if delusions also make a positive contribution with regard to eudaimonic wellbeing, although we did see that in some studies elaborated delusions have been shown to increase people's sense of coherence, which suggests that they feel their lives have a purpose. In other cases, it is not clear what contributions delusions make. Do delusions in schizophrenia and OCD ameliorate people's functioning? Answering such a question requires weighing one effect against the other. Relief from anxiety and the restoration of intrapsychic coherence support good functioning, but delusions are very often all‐absorbing, a feature which seems to speak in favor of the common belief that delusions interfere with good functioning.

One way to establish if and to what extent delusions are conducive to good functioning in the critical circumstances in which they emerge would be to compare indicators of good functioning in people who experience mental health issues and have delusions with people who experience similar mental health issues but have no delusions. Given that both groups, with and without delusions, are vulnerable to the similar psychological difficulties, evidence for the psychological adaptiveness of delusions would lie in whether people with delusions function better than their people without. What we know about delusions at this stage suggests that the prospects for delusions to enhance good functioning are not promising.

## 8 CONCLUSION

In this paper, we reviewed some of the arguments in favor of clinical delusions having psychologically adaptive or biologically adaptive features. Whereas the costs of delusions are well‐known—and many of the definitions of delusions are indeed based on their negative features—the potential benefits of delusions have not been studied thoroughly yet. We highlighted both interesting contributions and significant gaps where plausible claims need to be investigated further. It is safe to conclude at this stage that many delusions are in the long run psychologically harmful and biologically maladaptive, but that their adoption can be understood in context as offering some short‐term benefits, as a response to an emergency situation, although different authors characterize the nature of the emergency and the response to it in different terms. For instance, some motivated delusions can be seen as a coping mechanism to avoid major depression and suicide in case of overwhelming negative emotions caused by trauma; delusions formed in response to anomalous experiences can be seen as a means of relieving anxiety and restoring habitual processes of learning which were disrupted by aberrant prediction‐error signals; and delusions in OCD and MDD can be seen as reducing the conflict caused by a clash in the person's emotions, beliefs, and behaviors and as restoring some levels of intrapsychic coherence (see Table 1).

**Table 1:** Sample reasons for and against the adaptiveness of delusions found in the literature

	Wellbeing and good functioning	Survival, good health, and reproduction
Delusional beliefs in schizophrenia	Their adoption brings short‐term anxiety relief by ending the uncertainty caused by perplexing experience. Depending on their content, their maintenance may cause distress. They are also likely to compromise socialization due to the lack of a shared reality.	According to some predictive processing accounts, their adoption helps resume automated learning after disrupted prediction‐error signals. Habitual processes enable engagement with the physical and social environment. Given that flexible learning relative to the delusional content is disabled, delusions are fixed beliefs.
Delusional beliefs in dementia	Their adoption and maintenance may lead to the construction of a better self and a better reality or fill explanatory gaps created by memory impairments. Their maintenance may cause inconsistencies in the self‐narrative and compromise socialization due to the lack of a shared reality.	They may contribute to self‐esteem and reduce the risk of depression. They may also support the pursuit of one's goals by sustaining motivation. By distorting reality, they may prevent one from finding the best means to achieve one's goals.
“Motivated” delusions	Their adoption and maintenance allow one to construct a better self and a better reality in response to negative emotions that could otherwise become overwhelming. Clashes between delusional content and reality may cause confusion and disappointment and compromise social relationships due to lack of a shared reality.	By making one feel better, they enhance mood and reduce the risk of depression. They may also support the pursuit of one's goals by sustaining motivation. Due their distorting reality, they may prevent one from finding the best means to achieve one's goals.
Delusional beliefs in MDD or OCD	They restore coherence between low mood and belief (MDD); and between obsessive and compulsive behavior and belief (OCD). Thus, they reduce fragmentation in the self‐narrative and tension in the sense of self. They can contribute to symptoms of MDD or OCD worsening.	By making one feel worse, more guilty and inadequate, and less competent, delusions in MDD adversely affect mood which may negatively impact on goal pursuit via motivation. People with OCD who have delusions show poorer functioning and higher levels of depression than people with OCD who do not have delusions.

There are several questions that the empirical and philosophical literature has as yet failed to answer satisfactorily, and these are relevant to the adaptiveness of delusions. One is whether it makes sense to talk about delusions (and other beliefs regarded as symptoms of psychiatric disorders) as *pathological* in themselves, or whether their role should be evaluated only as part of a more comprehensive analysis of a person's behavioral patterns. If delusions are pathological, what makes them so? Is it simply the fact that they are the outcome of a malfunction, or is the fact that they have detrimental effects on people's agency and survival? Answering such a question sounds daunting, but it is important to consider the implications that claims about the adaptiveness of delusions have for clinical practice, science, and society at large. A change of perspective in the way delusions are defined and assessed would also bring new levels of complexity to both symptom management in psychiatry and cognitive models in cognitive neuroscience research. Further, and most importantly, viewing delusions as responses to a crisis and as imperfect solutions to serious problems may be instrumental to challenging the pervasive stigma still associated with psychotic symptoms. An appreciation of the role that the adoption of a delusional belief may play should inform interactions between people who experience delusions and people who do not, hopefully enhancing understanding and cooperation.

## CONFLICT OF INTEREST

The authors have declared no conflicts of interest for this article.

## RELATED WIREs ARTICLES

https://doi.org/10.1002/WCS.121

https://doi.org/10.1002/wcs.1339
